# Do archery athletes’ hand dexterity, strength, and cognition exceed those of age-matched athletes and non-athletes? A cross-sectional study

**DOI:** 10.3389/fphys.2026.1880932

**Published:** 2026-07-15

**Authors:** Xinpeng Gao, Fuxin Hong, Jinlong Hou, Yanan Gao, Qianqian Han, Ming Li

**Affiliations:** 1 School of Physical Education, Yulin University, Yulin, China; 2 Department of Physical Education, Pukyong National University, Busan, Republic of Korea; 3 Department of Physical Education, Faculty of Sport and Leisure, Guangdong Ocean University, Zhanjiang, China; 4 College of Education and Sports Sciences, Yangtze University, Jingzhou, China

**Keywords:** archery athletes, basketball athletes, cognitive function, fine motor skills, handgrip strength, sport-specific adaptation

## Abstract

**Purpose:**

Sport-specific training may reshape athletes’ executive function and manual fine-motor control in a discipline-specific manner, affecting the efficiency of sensorimotor-cognitive coupling. Open-skill and closed-skill sports differ in environmental uncertainty and movement stability demands, but direct comparisons of hand dexterity, inhibitory control, and their interrelations across these sport types are limited.

**Methods:**

In this cross-sectional observational study, 114 participants (17 females, 97 males; mean age 20.07 ± 1.64 years) were recruited into three groups: Basketball athletes (BA), Archery athletes (AA), and Sedentary controls (SC). Under a unified testing protocol, participants completed the Purdue Pegboard Test (PPT), Grooved Pegboard Test (GPT), Handgrip strength assessment, the Montreal Cognitive Assessment (MoCA), and the Stroop Color-Word (SCW) Test to evaluate unilateral and bilateral fine manual dexterity, hand strength, global cognition, and inhibitory control.

**Results:**

AA outperformed both BA and SC on fine-motor tasks (PPT and GPT; all p < 0.05; d = 0.76–0.96 vs. SC). BA showed significantly greater grip strength and higher MoCA total scores than AA and SC (all p < 0.05; grip strength d = 0.95 and MoCA d = 0.83 vs. SC). Both athletic groups exceeded SC on PPT, GPT, and grip strength measures (all p < 0.05). On Stroop measures, BA showed higher word-reading completion than SC and AA, whereas AA scored lower than both SC and BA under the color-naming and color-word interference conditions (all p < 0.05); error counts did not differ between groups.

**Conclusion:**

Findings indicate sport-specific adaptations: archery training is associated with superior fine motor control and movement stability, whereas basketball training is linked to enhanced neuromuscular strength and higher global cognitive screening scores (MoCA), together with better performance on a Stroop interference task. These specialization effects suggest that inter-sport assessments and training programs should account for discipline characteristics and integrate targeted strength and cognitive training to promote more balanced athlete development. Given the cross-sectional design, these associations cannot establish causality, as self-selection into specific sports cannot be distinguished from training-induced adaptation; the sex-imbalanced sample (97 males, 17 females) further limits generalizability. Longitudinal or intervention studies are warranted.

## 1 Introduction

Fine motor ability is a fundamental basis for athletic performance and independence in daily functioning. Manual dexterity is defined as an individual’s capacity to exert high-precision spatiotemporal control and force modulation of finger–hand movements under task constraints. This capacity depends on several mechanisms acting together: visuomotor integration, the use of sensory feedback, and internal model-based prediction. Together, these allow movements to be monitored and corrected online (Issurin, 2010; Wolpert et al., 1995; Krakauer and Mazzoni, 2011). As a significant behavioral indicator of neuromotor function, manual dexterity is utilized not only in clinical functional assessments but also to quantify the level of fine manipulation and upper-limb control (Mathiowetz et al., 1985). Furthermore, fine motor control is not merely a peripheral execution outcome but rather interacts profoundly with executive functions. Inhibitory control, attentional allocation, and error monitoring shape how accurately fine actions are carried out. They do so by guiding action selection, suppressing competing responses, and correcting movements online. Collectively, these processes constitute the core “cognitive–motor coupling system” of motor performance (Diamond, 2013; Cristofori et al., 2019).

Sport-specific training has been shown to differentially shape the cognitive–motor coupling system, such that distinct disciplines induce divergent adaptations in cognitive and motor control that are not solely attributable to overall activity volume (Gu et al., 2019). It is widely accepted that sports can be categorized according to environmental predictability and skill structure. In this classification system, sports are typically divided into two distinct categories: open-skill exercises (OSE) and closed-skill exercises (CSE). The occurrence of OSEs is characterized by dynamic and unpredictable contexts, emphasizing rapid information updating, response selection, and inhibitory control (Gu et al., 2019; Heilmann et al., 2022). In contrast, CSEs are typified by relatively stable environments, prioritizing self-paced timing, movement consistency, and error minimization (Heilmann et al., 2022). Systematic reviews and meta-analyses suggest that OSE and CSE athletes may differ in executive functions such as inhibitory control and cognitive flexibility. However, most existing studies examine only a single sport or a single cognitive domain. Few studies compare executive function and fine motor control within a single experimental framework, and this gap limits how well sport-specific training adaptations can be interpreted mechanistically (Gu et al., 2019; Heilmann et al., 2022).

The selection of basketball and archery as the contrasting sports for testing these hypotheses was deliberate. As a prototypical open-skill exercise (OSE), basketball requires athletes to continuously integrate visual cues within high-interference, rapidly changing competitive contexts, to perform bimanual coordination and fine manipulations under time pressure, and to suppress irrelevant or erroneous responses to minimize errors. Conversely, archery, a conventional closed-skill exercise (CSE), is performed in a relatively static environment that prioritizes movement repeatability and stability, emphasizing sustained attention, reduction of movement variability, and precise force modulation (Wang et al., 2013). These two sport types emphasize different demands: adaptation to external uncertainty (OSE) versus self-paced stability (CSE). We therefore propose that OSE training preferentially enhances conflict inhibition and rapid response control, whereas CSE training preferentially promotes movement consistency, fine force control, and sustained attention (Wang et al., 2013; Heilmann et al., 2022).

Although there is some evidence from studies of athletes that support the hypothesis that there is a link between executive functions and sporting performance (Vestberg et al., 2012), there is a paucity of empirical studies that directly compare manual dexterity and cognitive function in basketball and archery athletes. Furthermore, there is a dearth of studies that make concurrent assessments of the cognitive–fine motor coupling. In order to address this gap, the present study employs a multifaceted approach to assess hand motor performance (measured using the GPT, PPT, and maximal handgrip strength) and cognitive function (assessed using the SCW Test and the MoCA). This comprehensive approach enables the characterization of differential expressions of the cognitive–motor system under open- versus closed-skill training backgrounds. It is hypothesized that archers will demonstrate superior performance on hand-dexterity measures that emphasize movement consistency and fine force control, whereas basketball players will demonstrate superior performance on executive-function measures related to conflict inhibition. This study has two aims: first, to separate the general effects of physical activity from sport-specific training adaptations; and second, to provide an empirical basis for designing targeted cognitive–motor interventions. In doing so, the study aims to inform athlete selection and training optimization.

## 2 Materials and methods

### 2.1 Participants

The present study was conducted from January to February 2026, with participant recruitment and screening being carried out in China. The research team conducted targeted recruitment by telephone contact with coaches and school teachers and completed preliminary eligibility screening; a total of 120 participants were enrolled. Prior to enrolment, written informed consent was obtained from all participants. The study was conducted in accordance with the Declaration of Helsinki and received approval from the Ethics Committee of the School of Education and Physical Education at Yangtze University in China (Ethics approval no.: CESS−20260123003; approved on 2026−01−23). The inclusion criteria comprised the following: (a) age 18–22 years; (b) BMI 18.5–23.9 kg/m²; (c) regular diet and sleep over the preceding 3 months (7–9 hours of sleep per night, with no long−term dieting or binge eating); (d) no congenital diseases, recent sports injuries, or chronic diseases, and no diagnosed psychiatric or neurological disorders; (e) no long−term use of medications likely to affect physical function; (f) non−smokers with normal or corrected−to−normal vision; and (g) clearance to participate in physical exercise without discomfort or health risk as assessed by the Physical Activity Readiness Questionnaire (PAR−Q). It is evident that both sports had attained provincial or national competitive levels, and the athletes had participated in provincial or national first-level leagues. Moreover, they had undergone at least six months of intensified training prior to participation. The training characteristics of the two athlete groups are outlined below: The mean training experience of the BA group was 9.8 years (± 2.2 years), and the mean daily training time was 3.50 hours (± 1.08 hours). The mean training experience of the AA group was 9.4 years (± 2.5 years), and the mean daily training time was 4.00 hours (± 1.23 hours). SC group was defined as engaging in less than 30 minutes per week of moderate-intensity physical activity and having not regularly participated in sports in the past three years. Exclusion criteria encompassed neurological disorders or learning disabilities; limb injuries within six months prior to assessment that could affect testing; and any cognitive impairment or other condition likely to influence measurement outcomes. Prior to the commencement of the formal experiment, all participants completed questionnaires to collect data on age, sporting discipline, consecutive years of training, medical history, handedness and writing laterality. They also underwent screening with the PAR-Q. Sample size estimation was conducted using GPower 3 via an *a priori* power analysis (using GPower 3.1.9.2; α = 0.05; 1−β = 0.80; effect size f = 0.35, based on pilot data), which indicated at least 111 participants were required; 120 were ultimately recruited to maximize statistical power and mitigate potential attrition. The actual sample comprised 114 participants, and *post hoc* analysis indicated a statistical power of 0.92.

### 2.2 Experimental procedures

The study employed a cross-sectional design, with all measurements completed during a fixed daily time window (10:00–16:30) to control for diurnal effects. Each participant attended two visits: an initial visit for a practice session to familiarize participants with the procedures, anthropometric measures (height and weight), and explanation of study-related risks; and a second visit 48 hours later to complete the five formal assessments, with the assessment order randomized and counterbalanced. The formal assessments comprised two hand−dexterity tests (the Grooved Pegboard Test and the Purdue Pegboard Test), two cognitive measures (the Montreal Cognitive Assessment and the Stroop Color–Word Test), and a handgrip strength test. Prior to each test, a practice trial was conducted, with each assessment session lasting approximately 35–40 minutes. Testing was conducted in a provincial laboratory situated in proximity to the sports club. In an effort to minimize the potential for bias resulting from disparities in training background, basketball and archery athletes were recruited from the same sports club and training camp. This approach was adopted to ensure that the training environment and intensity were comparable; the sedentary control group was recruited from nearby universities and matched for age and lifestyle.

### 2.3 Hand dynamometer

Handgrip strength was measured using a Jamar 5030J1 hydraulic dynamometer. Testing posture strictly followed the standardized requirements recommended by the American Society of Hand Therapists (ASHT): participants were seated with the shoulder adducted and neutrally rotated, the elbow flexed at 90°, the forearm in a neutral position, and the wrist maintained in a neutral to 0°–30° dorsiflexion position. The testing protocol involved 3 repeated trials per hand, with each trial consisting of a maximal isometric contraction sustained for 6 seconds. A 60−second rest interval was provided between trials to avoid muscle fatigue. The final handgrip strength value was calculated as the mean of the 3 measurements. This method has been validated to yield superior test–retest reliability compared to single trials or the maximum of three trials, particularly showing stability in young female populations (Mathiowetz et al., 1984).

### 2.4 Grooved pegboard test

The finger-dexterity assessments were administered using the standardized GPT protocol (Marmon et al., 2011). The experimental phase of the study was conducted with the participant’s dominant hand. The task required the insertion of 25 slotted pegs into a 5 x 5 pegboard as expeditiously as possible. Participants who were right-handed commenced from the top-left corner and proceeded in rows from left to right and top to bottom. Participants who were left-handed initiated from the top-right corner and proceeded in rows from right to left and top to bottom. Prior to insertion, it was imperative that each peg be meticulously aligned with the slot, ensuring congruence with the board groove. The procedure comprised one practice trial to familiarize the participant with the movement, followed by three formal trials for each hand. The raw score was defined as the total time from the examiner’s “start” signal to the insertion of the final peg (Wang et al., 2024).

### 2.5 Purdue pegboard test

The Purdue Pegboard is a standardized instrument utilized to evaluate finger and hand dexterity. It is frequently employed in occupational therapy, motor coordination, and neuropsychological assessments to identify and monitor impairments in neurological, musculoskeletal, and motor function. The experimental phase was conducted in an environment that was both quiet and devoid of any potential disturbances. The apparatus under consideration consists of a board with two columns of 25 holes each, featuring four grooves for placing pins, washers, and collars. The pins were placed inside trays, while washers and collars were in the central tray. The test is composed of four subtests: The experiment comprised four stages. (1) The subject was required to insert as many pins as possible into the corresponding column using either the dominant or non-dominant hand, within a time limit of 30 seconds. (2) The subject was required to insert pins into aligned holes using both hands simultaneously, within a time limit of 30 seconds. (3) The subject was required to alternately use the left and right hands to assemble components (pin + washer + collar + washer) as many times as possible within a time limit of one minute. A 15-second rest period was allocated between each task, as well as after the completion of each task. Prior to the commencement of formal testing, participants were provided with practice trials. The entire task block was repeated on three occasions, with a total duration of approximately 12 minutes. The tester continuously observed and recorded the number of pins inserted and the completion time for each task, with final scores calculated as the mean of three repetitions for each subtest. The test design was non-stressful, with participants able to request clarification or brief breaks to ensure data validity and safety (McLellan, 2011; Chen et al., 2024).

### 2.6 Stroop color−word test

Given the established influence of cognitive function on GPT performance in finger-dexterity tests (Tolle et al., 2020), the present study adopted the Stroop paradigm established by Periáñez et al. to focus on cognitive performance under the SCW conflict condition (Periáñez et al., 2021). The participants were assigned to one of three tasks: Stroop Word-Reading (SWR): items consisted of the words “red,” “green,” and “blue” printed in black ink, arranged randomly in vertical columns with no repeated word within the same column; participants were instructed to read the word meaning. Stroop Color-Naming (SCN): stimuli comprised four different strings printed in red, green, or blue ink, and participants were instructed to name the ink color; the stimulus sequence was arranged to avoid immediate repetition of color and word order with the SWR block. In the SCW task, the word meaning and ink color were mismatched. Participants were instructed to ignore the word meaning and report only the ink color. This was done to elicit the color-word interference effect. It is evident that all tasks were completed immediately after the GPT. The score for each task was determined by the number of correctly answered items within 45 seconds, and these scores were used to reflect cognitive differences among participant groups (Wang et al., 2024).

### 2.7 Montreal cognitive assessment

The MoCA was administered as one of five tests during the subject’s second visit, in a randomized, balanced sequence. All formal assessments were conducted after the pre-test had been completed and participant status had been confirmed. The MoCA was administered face-to-face by assessors who had received uniform training, using the Chinese paper version that had been validated for use with the Chinese population (Chen et al., 2016), in accordance with standard operating procedures. The process included no practice runs, took approximately 10 minutes and required verification of the audio-visual conditions prior to administration, with any assistive devices being prohibited. All participants were assessed in quiet, independent testing rooms at a provincial laboratory near the club. Scoring was completed immediately and reviewed by a second researcher. Raw data were entered into the database and stored in a de-identified manner on the same day. Participants could take breaks if they felt any discomfort, and notes were recorded to ensure assessment consistency and data quality.

### 2.8 Statistical analysis

Analyses were performed using SPSS 26.0 (IBM, Armonk, NY, USA) and GraphPad Prism 9.0.0 (GraphPad Software, San Diego, CA, USA), and the results were cross-checked to enhance the robustness of the statistical conclusions. Continuous variables are presented as the mean ± standard deviation (SD). Normality was assessed using the Shapiro–Wilk test, and homogeneity of variance was assessed using Levene’s test. If the assumptions of normality and homogeneity were both met, group differences were examined using one-way ANOVA and the effect size, η², was reported and interpreted as follows: <0.01 (very small), 0.01–0.06 (small), 0.06–0.14 (medium) and >0.14 (large). If the homogeneity of variance assumption was violated, a Welch-corrected one-way ANOVA was used instead. *Post hoc* pairwise comparisons used Tukey’s test when variances were homogeneous and Games–Howell’s test when they were not. Pairwise effect sizes were reported as Cohen’s d and interpreted as |d| < 0.2 (small), 0.2 ≤ |d| < 0.8 (moderate) and |d| ≥ 0.8 (large). All tests were two-tailed, with significance set at P < 0.05. To facilitate the evaluation of practical significance, the results report key test statistics together with the corresponding effect sizes.

## 3 Results

### 3.1 Purdue pegboard test

**Figure 1:**
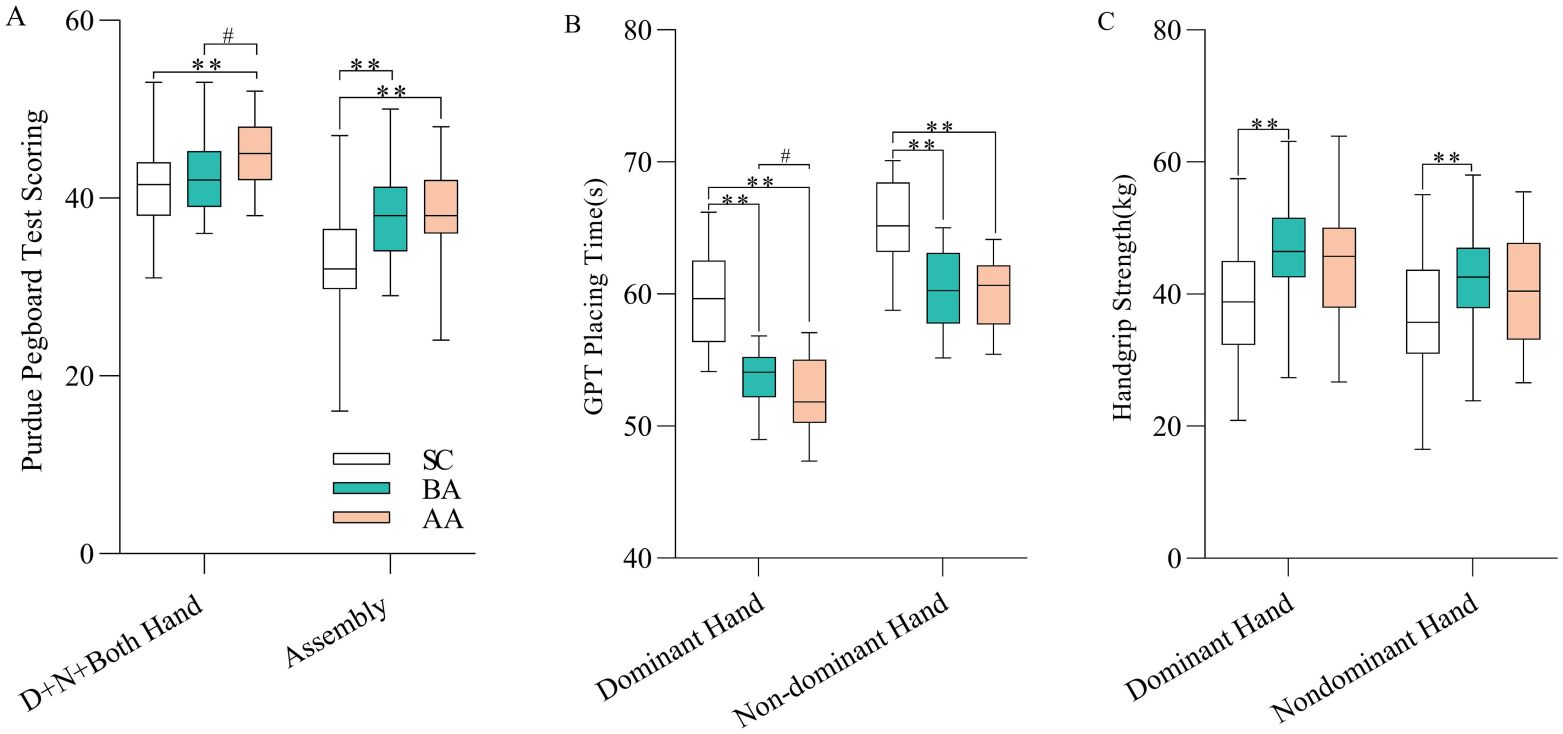
Intergroup differences were analyzed using one-way analysis of variance (ANOVA), followed by Tukey’s post hoc test. Abbreviations: **(A)** represents the Purdue Pegboard test scoring, **(B)** represents the placing time on the grooved pegboard test, and **(C)** represents handgrip strength. GPT, grooved Pegboard Test; SC, Sedentary Control; BA, Basketball athletes; AA, archery athletes; D, dominant hand; N, non-dominant hand; D+N+Both hands, dominant hand, non-dominant hand, and both hands combined. *P < 0.05, **P < 0.01 vs.SC; #P < 0.05, ##P < 0.01 vs. BA; no symbol indicates P > 0.05.

The dimensions of the Purdue Pegboard were found to conform to the normal distribution (p > 0.05) and homogeneity of variance, thus necessitating the implementation of a one−way ANOVA with Tukey HSD *post hoc* tests. The dominant hand was found to have mean values of 15.16 ± 1.93, 15.84 ± 1.83, and 16.61 ± 1.27 for SC, BA, and AA, respectively; F = 6.717, p = 0.002. A was found to be significantly higher than SC (p < 0.001, d = 0.88) and BA (p = 0.040, d = 0.48). Conversely, BA did not differ significantly from SC (p = 0.121, d = 0.36) (**Figure 1C**; **Table 1**). The mean values obtained for the non-dominant hand were 13.87 ± 1.92, 14.39 ± 1.86, and 15.32 ± 1.78, respectively. A one-way analysis of variance (ANOVA) was conducted, and the resultant F value was 5.780, with a p value of 0.004. A significantly higher mean value was observed for AA in comparison to both SC (p = 0.001, d = 0.78) and BA (p = 0.033, d = 0.51). Conversely, BA did not differ significantly from SC (p = 0.235, d = 0.27) (**Figure 1C**; **Table 1**). Bilateral coordination: mean values were 11.74 ± 1.48, 12.16 ± 1.53, and 12.84 ± 1.23 respectively; F = 6.327, p = 0.003. AA was significantly higher than SC (p = 0.001, d = 0.80) and BA (p = 0.037, d = 0.49); BA showed no significant difference from SC (p = 0.233, d = 0.28) (**Table 1**). Dominant hand + Non-dominant hand + Both hands total (D + N + both hands total): The mean scores were 40.76 ± 4.61, 42.39 ± 4.18 and 44.76 ± 3.66, respectively. F = 8.605, p < 0.001. AA significantly exceeded SC (p < 0.001, d = 0.96) and BA (p = 0.012, d = 0.60), but there was no significant difference between SC and BA (p = 0.115, d = 0.37) (see **Table 1**). The combined scores had mean values of 33.13 ± 6.97, 37.92 ± 4.38 and 37.82 ± 5.07, respectively (F = 8.883, p < 0.001). Both BA and AA were significantly higher than SC (BA vs. SC: p < 0.001, d = 0.82; AA vs. SC: p = 0.002, d = 0.76). No difference was observed between BA and AA (p = 0.924, d = 0.02) (**Table 1**).

**Table 1:** Purdue pegboard test.

Purdue Pegboard test	SC (n=38)	BA (n=38)	AA (n=38)	Overall F	P/η²
Dominant Hand Scoring	15.16 ± 1.93	15.84 ± 1.83	16.61 ± 1.27**#	6.717	0.002/0.108
Nondominant Hand Scoring	13.87 ± 1.92	14.39 ± 1.86	15.32 ± 1.78**#	5.780	0.001/0.094
Both Hand Scoring	11.74 ± 1.48	12.16 ± 1.53	12.84 ± 1.23**#	6.327	0.003/0.102
D+N+Both Hand Scoring	40.76 ± 4.61	42.39 ± 4.18	44.76 ± 3.66**#	8.605	<0.001/0.123
Assembly Scoring	33.13 ± 6.97	37.92 ± 4.38**	37.82 ± 5.07**	8.883	< 0.001/0.138

Inter-group differences were analyzed by one-way ANOVA with post-hoc Tukey test. Group abbreviations: SC, Sedentary controls; BA, Basketball athletes; AA, Archery athletes. *P < 0.05, **P < 0.01 vs.SC; ^#^P < 0.05, ^##^P < 0.01 vs. BA; no symbol indicates P > 0.05.

### 3.2 Grooved pegboard test

The Grooved Pegboard Test used completion time as the outcome measure (**Figure 1B**). For the dominant hand, the data met the normality criterion but violated the homogeneity of variance criterion, so a one-way ANOVA with Welch correction was applied. There was a highly significant difference among the three groups (SC, BA and AA: 59.54 ± 3.37, 53.84 ± 1.93 and 52.41 ± 2.87, respectively; F = 67.848, p < 0.001). *Post hoc* comparisons revealed that the AA and BA groups completed the task significantly faster than the SC group (both p < 0.001, d = 2.27 and 2.08, respectively) and that the AA group was faster than the BA group (p = 0.014, d = 0.58). Data for the non-dominant hand satisfied both normality and homogeneity of variance criteria. One-way ANOVA with Tukey HSD *post hoc* analysis revealed highly significant group differences (65.71 ± 2.82, 60.29 ± 2.78, 60.26 ± 2.34; F = 51.541, p < 0.001). The AA and BA groups completed the task significantly faster than the SC group (both p < 0.001, d = 2.10 and 1.93, respectively), but there was no significant difference between the two sports groups (p = 0.917, d = 0.01).

### 3.3 Handgrip strength test

All grip strength measures met the criteria for normality and homogeneity of variance. A one-way ANOVA revealed a significant difference between groups for dominant-hand grip strength (SC, BA and AA: 38.8 ± 9.38, 46.98 ± 7.66 and 43.98 ± 9.35, respectively; F = 8.845, p < 0.001). Tukey HSD *post hoc* tests revealed that the BA and AA groups exhibited significantly greater dominant-hand grip strength than the SC group (p < 0.001 and p = 0.015, respectively; d = 0.95 and d = 0.55), though no significant difference was observed between BA and AA (p = 0.106, d = 0.35). Group differences were also observed for the non-dominant hand (35.71 ± 9.64, 42.52 ± 7.51, 40.60 ± 8.70; F = 6.303, p = 0.003). *Post hoc* tests showed that the grip strength of the non-dominant hand in the BA and AA groups was both significantly higher than in the SC group (p = 0.001, d = 0.78; p = 0.033, d = 0.53), with no significant difference between the BA and AA groups (p = 0.211, d = 0.24) (**Figure 1C**).

### 3.4 Cognitive function tests

The cognitive test results are shown in **Table 2**. MoCA scores were analyzed using a one-way ANOVA with Welch correction and were found to differ significantly between groups (SC: 27.29 ± 1.68; BA: 28.50 ± 1.18; AA: 26.87 ± 2.74; F = 6.978, p = 0.001). *Post hoc* comparisons revealed that the BA group performed significantly better than the SC group (p < 0.001, d = 0.83) and the AA group (p = 0.001, d = 0.77), while there was no significant difference between the SC and AA groups (p = 0.422, d = 0.18).

**Table 2:** Cognitive function assessment.

Parameter		SC (n=38)	BA (n=38)	AA (n=38)	Overall F	P/η²
MoCA		27.29 ± 1.68	28.50 ± 1.18**	26.87 ± 2.74#	6.978	0.001/0.112
SCWT	SWR	106.66 ± 16.50	116.42 ± 18.60**	108.08 ± 19.75#	3.146	0.047/0.054
	SWR Error Count	1.29 ± 1.37	1.32 ± 1.56	1.87 ± 1.95	1.501	0.227/0.026
	SCN	73.71 ± 12.04	76.34 ± 9.72	68.21 ± 11.27 *#	5.357	0.006/0.088
	SCN Error Count	1.71 ± 1.49	1.63 ± 1.28	1.58 ± 1.54	0.080	0.923/0.001
	SCW	48.13 ± 12.11	48.00 ± 8.31	42.76 ± 7.59*#	3.909	0.023/0.066
	SCW Error Count	2.29 ± 1.77	2.50 ± 1.52	2.53 ± 1.98	0.205	0.815/0.004

Inter-group differences were analyzed by one-way ANOVA with post-hoc Tukey test. Abbreviations: MoCA, Montreal Cognitive Assessment; SCWT, Stroop Color Word Test; SWR, Stroop Word Reading; SCN, Stroop Color Naming; SCW, Stroop Color-Word; SC, Sedentary controls; BA, Basketball athletes; AA, Archery athletes. *P < 0.05, **P < 0.01 vs.SC; #P < 0.05, ##P < 0.01 vs. BA; no symbol indicates P > 0.05.

The stroop word reading (SWR) dimension of the SCW Test showed a marginally significant group difference (106.66 ± 16.50, 116.42 ± 18.60, 108.08 ± 19.75; F = 3.146, p = 0.047), with BA being higher than both SC and AA (p = 0.022, p = 0.049; d = 0.56, 0.43). SWR error counts did not differ between groups (F = 1.501, p = 0.227). The stroop color naming (SCN) dimension showed a significant difference (101.32 ± 10.56, 98.75 ± 11.23, 68.21 ± 11.27; F = 5.357, p = 0.006), with AA being lower than both SC and BA (both p < 0.05; d = 0.47, 0.77). SCN error counts showed no group difference (F = 0.080, p = 0.923). The stroop color-word (SCW) dimension also differed between groups (62.35 ± 8.12, 60.18 ± 7.89, 42.76 ± 7.59; F = 3.909, p = 0.020), with AA being lower than both SC and BA (p = 0.023 and p = 0.005, respectively; d = 0.53 and 0.66, respectively). SCW error counts likewise showed no significant group differences (F = 0.205, p = 0.815).

## 4 Discussion

This study examined patterns of difference and interrelationship between BA (OSE), AA (CSE) and SC populations in key abilities such as manual dexterity and inhibitory control. The main findings can be summarized as follows: (1) the AA group demonstrated superior manual dexterity (PPT and GPT); (2) the BA group exhibited greater grip strength and higher cognitive performance; and (3) both the BA and AA groups outperformed the SC group in terms of PPT, GPT, and strength measures. Overall, these results support the principle of task-specific adaptation, suggesting that long-term participation in sports with substantially different movement patterns, actions and cognitive demands leads to differentiated ability profiles (Sale, 1988).

The primary finding of this study is that archery athletes demonstrate superior performance in fine motor tasks that require high levels of manual dexterity, precision and submaximal force control. This aligns with archery-specific research indicating that ‘high-level archery performance relies on more stable posture control and finer output regulation’: archers must minimize body sway when aiming and releasing to reduce terminal output fluctuations and improve hit consistency (Ertan et al., 2003; Hayri Ertan, 2009). Furthermore, from a sport-specific perspective, previous reviews and empirical studies suggest that different sports may exhibit sport-specific differences in attention control and executive functions (OSE vs. CSE), providing broader contextual support for the notion that ‘sport-specific training shapes cognitive-motor control strategies’ (Voss et al., 2010; Jacobson and Matthaeus, 2014). However, it is important to emphasize that, while some findings from shooting sports (e.g. stability and fine control strategies) may provide indirect references for understanding common mechanisms in ‘high-precision aiming sports’, they should not be regarded as direct evidence for archery (Era et al., 1996).

According to human neuroscience research, long-term practice of fine motor skills is closely linked to experience-dependent neural adaptations. A classic study by Elbert et al. found that professional string instrumentalists had an enlarged cortical representation of their fingers, which demonstrates that intensive, long-term fine motor training can induce functional reorganization of the sensorimotor cortex (Elbert et al., 1995). Further complementary neuroimaging evidence supports the idea that even relatively short training periods can lead to changes in brain structure (Draganski et al., 2004). While the present study did not directly evaluate brain structure or function, the superior fine motor performance exhibited by archers aligns with the well-established principles of training-induced plasticity ( (Elbert et al., 1995; Draganski et al., 2004). Moreover, precision-focused sports may reduce movement variability, commonly referred to as ‘motor noise’ (Faisal et al., 2008). The ability to suppress task-irrelevant variability and generate highly consistent movement patterns is critical for archery performance and may partly explain why training adaptations transfer to laboratory fine-motor tests. Importantly, such adaptations are clearly task-specific: archers did not demonstrate superior grip strength, which further supports the specificity of training effects (Sale, 1988). Nevertheless, as no neuroimaging or neurophysiological data were collected and the design is cross-sectional, these neural interpretations remain speculative and should be regarded as hypotheses for future confirmation rather than direct evidence of training-induced cortical reorganization in the present sample.

Basketball athletes demonstrated significantly greater grip strength than archers and non-sporting participants. This finding is consistent with the view that hand grip strength is a better indicator of overall upper limb and whole body strength, as well as long-term load exposure, than of specific fine motor skills (Wind et al., 2010; Bohannon, 2008). It also aligns with reviews indicating that athletes in sports emphasizing contact, gripping and strength tend to demonstrate greater grip strength (Cronin et al., 2017). In terms of sport-specific differences between basketball and archery, basketball training and competition involve frequent physical contact, contesting possession and high-tension situations. Many basketball players also undertake systematic, higher-intensity resistance training (e.g. upper-limb push/pull exercises). Such training produces the largest gains in maximal strength. These gains are thought to arise mainly from neural adaptations—such as changes in motor unit recruitment and discharge properties and improved muscle coordination—which appear as higher outputs on maximal-effort grip tests (Sale, 1988; Suchomel et al., 2016). By contrast, although archery requires high upper-limb stability and fine control, training emphasizes maintaining stability and fine-tuning under submaximal loads rather than repeatedly pursuing maximal force. Therefore, it is understandable that archers do not show an advantage in terms of maximal grip strength. Differences in grip strength should therefore be interpreted in light of sport-specific training load and resistance-training history. They should not be equated simply with fine motor ability or sport-specific technical skill (Wind et al., 2010).

BA demonstrated superior cognitive performance compared to AA and SC. This finding aligns with evidence suggesting that OSEs (open, dynamic, unpredictable environments) impose continuous demands on executive functions, which are essential for effective decision-making in complex sporting contexts (Diamond, 2013; Wang et al., 2013). Specifically, basketball players had higher overall cognitive scores (MoCA, adjusted for education) than archers and non-athletes, and performed better on the SCW test. They achieved higher completion rates in the SWR condition, while archers performed worse than the other two groups in the SCN and SCW interference conditions. Error rates did not differ significantly between groups. In this study, Stroop performance is reported as the number of correct responses under standardized conditions. Group differences therefore reflect mainly selective attention and interference-control efficiency, rather than a greater tendency to make errors (Diamond, 2013; Nasreddine et al., 2005; Stroop, 1935). This pattern matches the cognitive demands of open-skill sports such as basketball. Athletes must continuously filter irrelevant information, update decisions and inhibit inappropriate responses in dynamic environments (Diamond, 2013; Wang et al., 2013). By contrast, archery is performed in a stable and predictable setting that emphasizes sustained, internally directed attention and motor stability. Therefore, the transfer to laboratory tasks that emphasize rapid interference resolution may be limited (Wang et al., 2013), which helps to explain the poorer performance in the Stroop test in the AA group. Multiple reviews have confirmed that open-skill sports produce greater improvements in executive functions than closed-skill sports. Koch and Krenn found that elite open-skill athletes outperformed elite closed-skill athletes on working memory and cognitive flexibility tasks (Koch and Krenn, 2021). Open-skill exercise has been shown to benefit inhibition control and cognitive flexibility in children, adolescents and adults, shortening response times and reducing switch costs in flexibility tasks (Lai et al., 2024). Furthermore, Lai et al. reported that open-skill sports improve adults’ working memory accuracy more than closed-skill sports. Another review concluded that open-skill practice yields superior gains in certain cognitive functions (Gu et al., 2019). However, Chakraborty et al. reported no significant differences in cognitive task performance or heart rate variability (HRV) between the two sports, arguing that participation in sport in general enhances cognition, regardless of sport type (Chakraborty et al., 2023). Our findings more strongly support the former perspective and also show that archers performed worse than sedentary controls on certain cognitive tests (Stroop interference condition). We speculate that, beyond individual and sport-related differences, precision sports may to some extent limit rapid cognitive processing—through reduced motor noise and an internally focused, highly concentrated attentional state. Additionally, the MoCA cannot dissociate specific executive subcomponents, and the Stroop task reflects both interference control and processing efficiency. Future studies should use more domain-specific cognitive tasks and complementary measures to further elucidate the mechanisms by which different sports shape cognitive advantages.

Beyond sport−specific differences, both athlete groups outperformed the SC group on multiple measures, further supporting the association between regular physical activity and improvements in neuromuscular function. Exercise can promote brain health via multiple pathways, including enhanced neurotrophic signaling, improved cerebrovascular function, and increased synaptic plasticity (Cotman and Berchtold, 2002; Erickson et al., 2011; Hötting and Röder, 2013). Notably, aerobic and mixed−mode exercise interventions have been shown to improve memory in previously sedentary adults and to increase hippocampal volume (Erickson et al., 2011). Although this study cannot elucidate causal mechanisms, the superior performance of active participants—particularly those in open−skill sports such as basketball—is consistent with the extensive literature on the cognitive and neural benefits of habitual physical activity (Cotman and Berchtold, 2002; Erickson et al., 2011; Hötting and Röder, 2013). From an applied standpoint, structured supplementary programs targeting trunk and core stability have been shown to improve functional movement, balance, and performance in basketball players (Saeidizadeh et al., 2026; Wang et al., 2025), and core stability training incorporating martial-arts-based movements can enhance postural control and neuromuscular coordination (Ebrahimi et al., 2026); such targeted approaches may complement the discipline-specific profiles observed here and inform balanced training prescriptions across sports.

This study has several potential limitations.Firstly, the cross-sectional design prevents causal inference, making it difficult to determine whether the observed group differences are due to adaptations induced by long-term, sport-specific training or reflect pre-existing individual cognitive differences arising from self-selection into specific sports. Future longitudinal follow-ups and intervention studies are needed to clarify causality. Secondly, only general cognitive assessment tools were used: the MoCA is a global cognitive screening instrument rather than a measure of executive function, and the Stroop test captures only one facet of inhibitory control, so domains such as working memory, cognitive flexibility, and planning were not assessed, which may limit sensitivity to sport-related cognitive functions. Thirdly, due to practical constraints, the sample size was relatively small. Fourthly, the sample was strongly male-dominated (97 males, 17 females), and sex differences in hand strength, dexterity, and cognitive performance may have influenced the findings; results should therefore be generalized with caution, and future studies should recruit sex-balanced samples. Finally, training history was characterized only by years of experience and mean daily training time, without detailed quantification of weekly training load and intensity, which should be reported in greater detail in future work. Future research should increase the sample size and combine comprehensive cognitive test batteries with neuroimaging techniques to further elucidate the mechanisms underlying sport-specific cognitive adaptations.

## 5 Conclusion

To our knowledge, this is the first study to compare AA, BA and age-matched SC in terms of hand fine-motor performance and cognitive outcomes. Our findings suggest that sport participation is associated with distinct performance profiles: AA demonstrate greater precision and stability in their movements, while BA exhibit superior neuromuscular strength and perform better on some measures of inhibitory control. These results suggest that generic physical or cognitive screening may be insufficient for comparing athletes across sports, and that complementary training approaches tailored to sport-specific demands may be warranted. However, because this study used a cross-sectional design, it is not possible to determine whether sport participation caused these differences or whether individuals with these characteristics self-selected into different sports. In addition, the cognitive findings should be interpreted cautiously, as MoCA reflects global cognition rather than executive function specifically. Longitudinal and intervention studies, with fuller control of training exposure, academic level, sex, and other relevant variables, are needed to confirm these associations and to evaluate their transfer to competitive performance.

## Data Availability

The raw data supporting the conclusions of this article will be made available by the authors, without undue reservation.
